# Supplementary material for: Evaluation of the Association between FGFR2 Gene Polymorphisms and Breast Cancer Risk in the Bangladeshi Population
Source: Genes (Basel). 2023 Mar 29;14(4):819. doi: 10.3390/genes14040819 (PMC10138231; doi:10.3390/genes14040819)
Supplement: Supplementary file 1 [file genes-14-00819-s001.zip › genes-2091318-supplementary.pdf]

Supplementary Materials: Figures S1-S3:

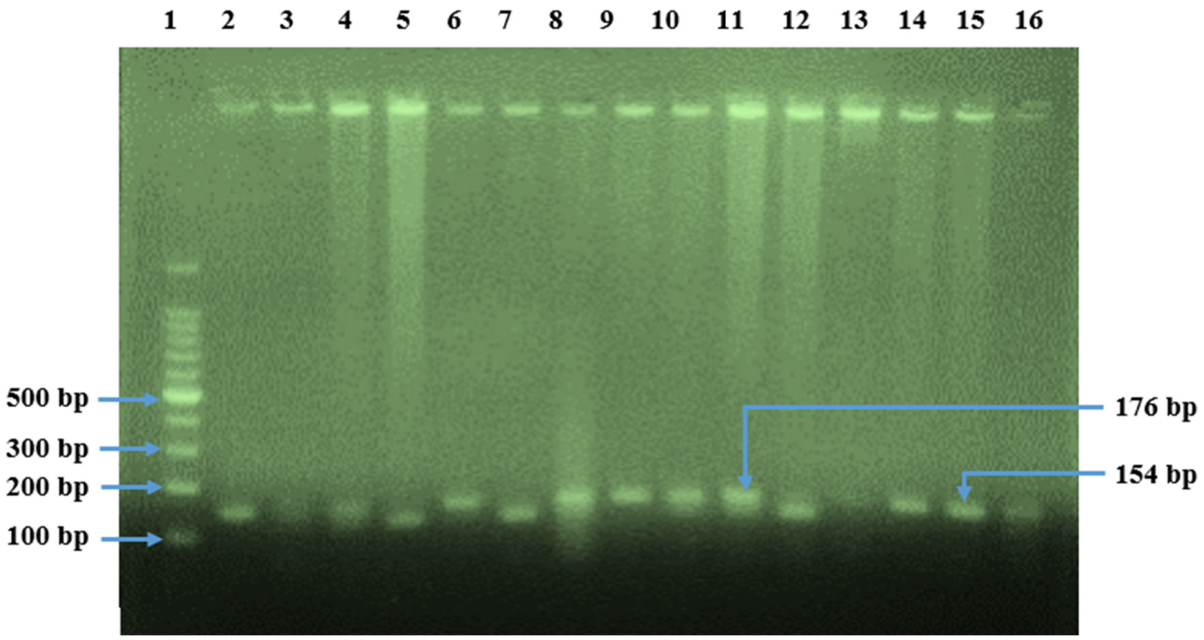

Figure S1: Restriction Endonuclease (HinP1I) digestion fragments of FGFR2 rs2981582 (1.3% agarose gel). Lane 1 indicates 100bp ladder; lanes 2-4,6,8,9 indicate TT genotype; lanes 10,11 indicate CT genotypes; lanes 5,7,12-16 indicate CC genotype.

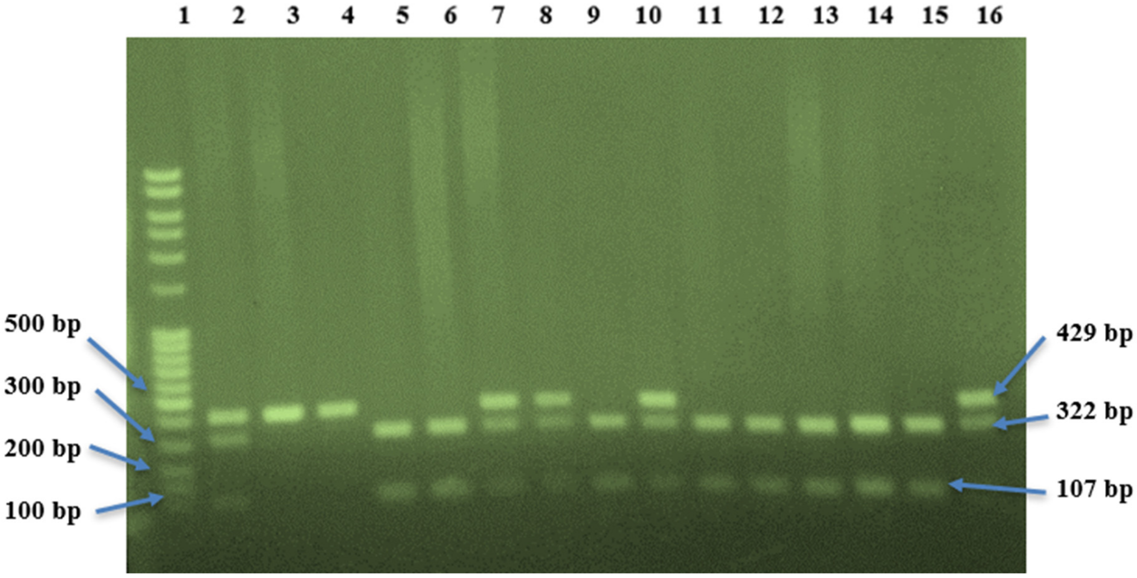

Figure S2: Restriction Endonuclease (HinP1I) digestion fragments of FGFR2 rs2420946 (1.3% agarose gel). Lane 1 indicates 100bp ladder; lanes 2,7,8,10,16 indicate CT genotype; lanes 3,4 indicate TT genotypes; lanes 5,6,9,11-15 indicate CC genotype.

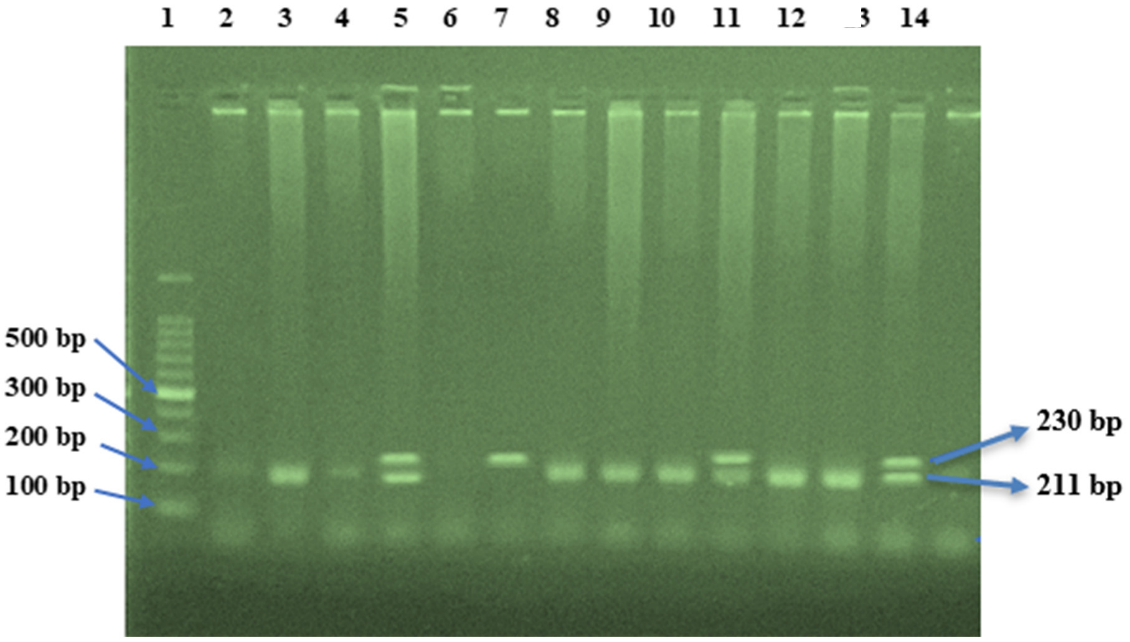

Figure S3: Restriction Endonuclease (HinP1I) digestion fragments of *FGFR2* rs1219648 (1.3% agarose gel). Lane 1 indicates 100bp ladder; lanes 2-4,8-10,12,13 indicate GG genotype; lanes 5,11,14 indicate AG genotypes; lanes 7 indicate AA genotype.
